# Delayed cerebrospinal fluid leak following lumbar interbody fusion caused by floating lamina: A case report

**DOI:** 10.1097/MD.0000000000046318

**Published:** 2026-05-12

**Authors:** Binbin Tang, Chen Chen, Han Zhang, Junwei Feng, Bing Wu, Zhongcheng An, Liqiang Dong

**Affiliations:** a Orthopedics and Traumatology II, The Second Affiliated Hospital of Zhejiang Chinese Medical University (Xinhua Hospital of Zhejiang Province), Hangzhou, China; b Orthopedics and Traumatology II, The Second Clinical Medical School of Zhejiang Chinese Medical University, Hangzhou, China.

**Keywords:** case report, delay cerebrospinal fluid leak, dural tear, floating lamina

## Abstract

**Rationale:**

Delayed cerebrospinal fluid (CSF) leakage after lumbar interbody fusion are scarce. Known causes include severe spinal canal stenosis and iatrogenic factors. This case presents a unique etiology, highlighting the importance of timely management. Prediction and prevention of postoperative complications is crucial.

**Patient concerns:**

We report a 37-year-old woman had recurrent low back pain and radiating right lower extremity pain for 2 years, which had exacerbated over the past week. Imaging revealed mild L5 spondylolisthesis, a free fragment of the L5-S1 lumbar disc, bilateral L5 pars defects (spondylolysis), and floating L5 lamina. Treatment involved L5 right laminectomy and L5-S1 lumbar interbody fusion. On postoperative day 6, the patient developed headache and bulging at the incision site.

**Diagnoses:**

Combined with analysis of the aspirated fluid, magnetic resonance imaging indications and patient’s symptoms, a diagnosis of delayed CSF leak was made.

**Interventions:**

Resection of the remaining and floating L5 left lamina and direct suturation were performed for dural tear repair followed by postoperative drainage tube placement.

**Outcomes:**

The patient was discharged with successful recovery 2 weeks after the second surgery. Follow-up lumbar magnetic resonance imaging confirmed resolution of the CSF leak and satisfactory recovery.

**Lessons:**

In lumbar pathologies with unique anatomy like a floating lamina, vigilance for delayed CSF leak is crucial. Meticulous preoperative imaging review and adaptable surgical strategies beyond conventional approaches are essential for preventing recurrence.

## 1. Introduction

Lumbar interbody fusion is a routine and effective spinal surgical procedure.^[1,2]^ It demonstrates safety and efficacy for conditions including lumbar disc herniation, lumbar spinal stenosis, spondylolisthesis, and degenerative lumbar scoliosis.^[3,4]^ Common complications include surgical site infection, implant loosening, cage subsidence, adjacent segment degeneration, and dural tears (DTs).^[5–8]^ DT leading to cerebrospinal fluid (CSF) leaks can be classified as either non-delayed or delayed.^[9]^ Non-delayed leaks manifest as symptoms immediately after surgery (within 5 days). Delayed leaks present with symptoms occurring more than 5 days after surgery.^[10]^ CSF leaks can be complicated by wound healing issues, infection risk, pseudomeningocele formation, low-pressure headaches, and, rarely, severe intracranial hemorrhage or brain herniation.^[8,9]^ Reportedly, the incidence of intraoperative DT causing CSF leaks in primary surgeries ranges from 2.6% to 8%, while delayed CSF leaks are even less common, with an incidence of only 0.83% to 14.3%. However, the probability of CSF leaks in lumbar revision surgeries can be as high as 13.2% to 21%.^[10]^

Diagnostic criteria for delayed CSF leaks include^[11]^:

- Symptoms suggestive of low CSF pressure (e.g., orthostatic headache, photophobia, nausea, and headache) appeared on postoperative day 5.
- Clear fluid drainage from the wound.
- Magnetic resonance imaging (MRI) confirmation of a DT and CSF leak.
- ±Positive β2-transferrin test.

Meeting the first 3 criteria, with or without the fourth, suggests delayed CSF leak.

This report describes a delayed CSF leak presenting on day 6 after surgery. The patient’s unique lumbar anatomy, a floating L5 lamina, was central to the leak’s development. This unusual etiology underscores how surgical experience with complex anatomy relates to outcomes.

## 2. Case presentation

A 37-year-old female working as a Pilates instructor and long-term resident in China, presented to our orthopedic department with a chief complaint of recurrent lower back pain for over 2 years, aggravated by right buttock radiating pain for 1 week. On admission, her visual analogue scale score was 6, and her Japanese Orthopedic Association score was 14. Physical examination revealed preserved spinal physiological curvature, tenderness over the L5/S1 interspace, radiating pain in the right buttock, and a positive straight leg raise test (50°) on the right side (negative on the left). Muscle strength assessment showed grade IV+ for the right great toe dorsiflexion and right ankle dorsiflexion, grade V for the right iliopsoas, and grade V for the left lower limb. A sensory examination revealed no numbness in the lower extremities. Peripheral circulation and physiological reflexes were intact bilaterally with negative Babinski signs.

**Figure 1.**
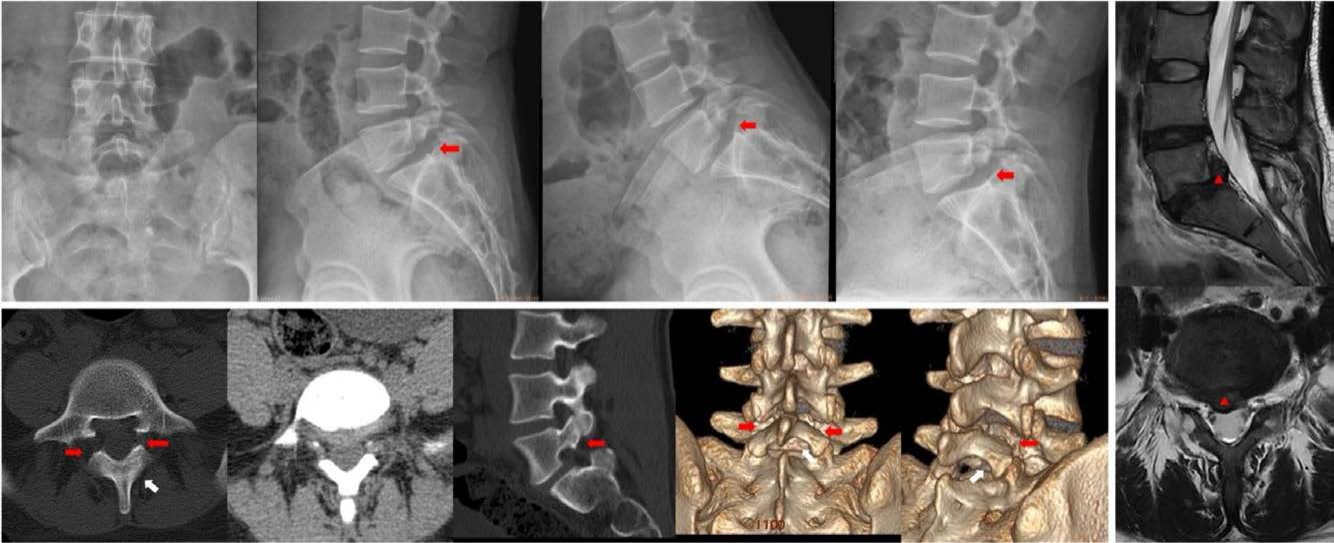
Preoperative radiological images. Red arrow: bilateral L5 pars defects (spondylolysis); white arrow: floating lamina; red triangle: herniated and free disc.

Lumbar anteroposterior, lateral, and flexion-extension radiographs indicated grade I spondylolisthesis at L5/S1 (according to the Meyerding classification^[12]^) with bilateral L5 pars defects (spondylolysis). Lumbar CT revealed complete instability of the bilateral L5/S1 facet joints and right-sided L5/S1 disc herniation compressing the S1 nerve root. A special structure-floating lamina was considered in this case. Lumbar MRI revealed a large, right-sided L5/S1 disc herniation with an upwardly migrated free fragment. Based on imaging findings and clinical symptoms, the patient met the criteria for lumbar interbody fusion surgery. The preoperative radiological images are shown in Figure 1.

**Figure 2.**
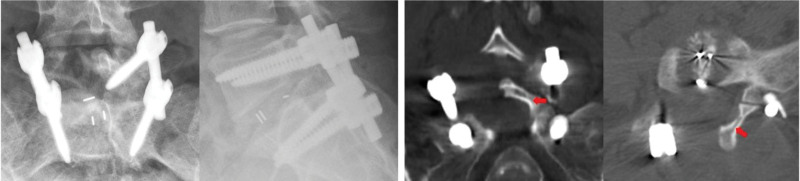
Postoperative radiological images. Red arrow: the residual left lamina but unstable.

The timeline of clinical changes and management is summarized in Table 1. The patient and her husband signed the informed consent of the operation. On the second day after admission, a conventional posterior lumbar interbody fusion^[13]^ was performed. This procedure involved pedicle screw instrumentation at L5 and S1, right L5 hemilaminectomy, and L5/S1 discectomy with interbody cage placement (Fig. 2).

**Table 1:** Timeline of condition change and clinical management.

Time point	Clinical manifestations	Management
Admission day	Low back pain radiating to the right buttock VAS score: 6; JOA score: 14	Preoperative examinations completed
Surgery day	–	L5/S1 PLIF, right L5 laminectomy, L5/S1 interspinous ligament tightening performed
Postoperative day 2	VAS score: 3; no radiating pain in lower limbs	Drainage tube removed; lumbar brace immobilization applied. Ambulated short distances
Postoperative day 6	Headache (VAS score: 5), aggravated by walking and bending, alleviated by lying flat	Clinical observation
Postoperative day 9	Headache absent while lying flat	Neurology consultation suspected intracranial hypotension. Administered high-volume fluid resuscitation and anti-infective therapy
Postoperative day 10	Headache absent while lying flat; low back pain present; headache markedly aggravated by lumbar pressure; surgical site swelling observed	Lumbar MRI revealed significant CSF leakage
Postoperative day 11	Headache absent while lying flat; headache triggered by minimal activity; surgical site swelling persisted	Bedside puncture drainage performed. Drainage yielded pale-yellow transparent fluid, laboratory analysis consistent with CSF
Postoperative day 12	Headache unrelieved; surgical site swelling persisted	Re-exploratory surgery performed; drainage tube placed in the surgical area
Post-reoperation day 1	Headache absent; no surgical site swelling	Fluid resuscitation continued; surgical site drainage maintained
Post-reoperation day 7	Headache absent; no surgical site swelling; fluid output ceased	Drainage tube removed
Post-reoperation day 15	Headache absent; surgical site healing well	Discharged uneventfully

CSF = cerebrospinal fluid, JOA = Japanese Orthopaedic Association, MRI = magnetic resonance image, PLIF = posterior lumbar interbody fusion, VAS = visual analogue scale.

No DT or CSF leakage occurred intraoperatively. We found the intact dural sac without any small tears. During the procedure, following a right hemilaminectomy for decompression, the residual left lamina was unstable. An interspinous ligament suture tightening procedure was performed to stabilize the lamina; however, this did not provide rigid fixation. The surgery was concluded with the patient reporting a visual analogue scale score of 3 and no lower extremity radiating pain. The surgical drain was removed on postoperative day 2 because of about 25 mL bloody liquid and sound skin of incision. The patient ambulated and performed simple activities while wearing the lumbar brace.

**Figure 3.**
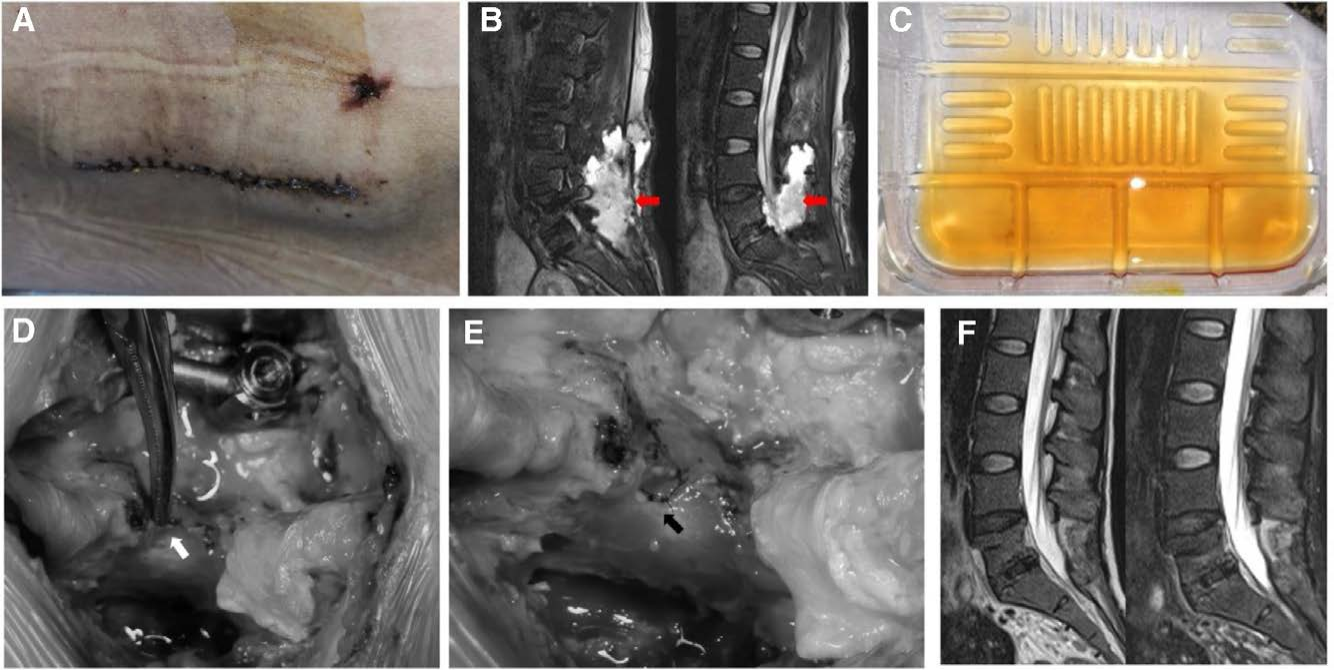
Clinical change and management of the case. (A) surgical site swelling; (B) lumbar MRI revealed significant cerebrospinal fluid (CSF) leakage; (C) drainage yielded pale-yellow transparent fluid, laboratory analysis consistent with CSF; and (D, E) intraoperative manifestation during the reoperation. (F) MRI images at follow-up. Red arrow: CSF leak in the MRI images. White arrow: the site of dural tear. Black arrow: the site of repair for dural tear. CSF = cerebrospinal fluid, MRI = magnetic resonance imaging.

On postoperative day 6, the patient complained of headache, predominantly frontal, radiating to the lumbar region. The headache worsened with walking and bending forward, and improved when lying flat. Analgesic medications were also administered. On postoperative day 9, the headache persisted without any relief. A neurological consultation was requested, recommending a brain MRI that showed no abnormalities. The neurologist suspected low-pressure headache and advised aggressive fluid hydration, antibiotic prophylaxis, and bed rest in the Trendelenburg position. At the beginning, we did not focus on the DT leading to headache. On postoperative day 10, the patient experienced significant lower back pain. The headache was markedly exacerbated by palpation of the lumbar region, and bulging was observed at the surgical site (Fig. 3A). Lumbar MRI revealed a large CSF collection suggestive of DT (Fig. 3B). On postoperative day 11, the patient was headache-free while supine but developed a headache with minimal activity. Bulging persisted at the surgical site. Bedside aspiration yielded clear, pale-yellow fluid. The analysis confirmed that the fluid was CSF (Fig. 3C). On postoperative day 12, the headache remained unrelieved and the surgical site continued to bulge. Considering the failure of conservative management, a second surgical exploration with drain placement was performed. Intraoperatively, a DT of approximately 3 mm in size was identified quickly and easily at the edge of the resected L5 lamina (Fig. 3D). The mobile left L5 lamina was deemed the cause; lumbar extension likely caused it to press ventrally against the dura, creating the tear. Consequently, residual left L5 lamina and spinous processes were completely removed. DT was repaired using interrupted 6 to 0 Prolene sutures. Dural closure was satisfactory, and no CSF leakage was observed (Fig. 3E). A 12-French silicone drain was placed at the surgical site.

On the first day after the second surgery, the patient was headache-free with no incision bulging. The drain was patent. There was no lower extremity radiating pain, and motor and sensory functions were normal. The drain output was recorded daily and gradually decreased. By the seventh day after the second surgery, the patient remained asymptomatic with no incision bulging and no drain output, prompting drain removal. Third-generation cephalosporin antibiotics were administered postoperatively for prophylaxis of infection. The patient developed diarrhea, but infectious causes were ruled out by stool culture. Antibiotics were subsequently discontinued and the surgical wound underwent regular dressing changes. Two weeks after the second surgery, the wound healed well and the patient was discharged uneventfully. Follow-up lumbar MRI confirmed resolution of the CSF leak and satisfactory recovery (Fig. 3F).

The patient shared the positive perspective on our treatment in the whole period in hospital. She had a strong compliance and an optimistic attitude. Close attention, meticulous care, good communication and determination to cure helped us solve this case in the end.

## 3. Discussion

This case presented with symptoms of low CSF pressure 6 days after lumbar interbody fusion. Subsequent lumbar MRI confirmed a CSF leak, which was classified as delayed CSF leak. A second surgical exploration successfully identified the cause of DT, which was repaired primarily with sutures. Postoperative drainage led to the resolution of symptoms and successful discharge.

The uniqueness of this case lies in the patient’s specific lumbar anatomy: bilateral L5 pars defects and complete instability of the L5/S1 facet joints constituting a floating L5 lamina. Repetitive stress likely contributed to the L5 spondylolisthesis and disc herniation with a free fragment. As a young female with significant occupational lumbar demands, the surgical strategy aimed to preserve normal lumbar structures. However, inherent instability of the unique anatomy is likely to predispose patients to DT. DT leading to CSF leakage is a common complication of spinal surgery. The incidence is higher in lumbar procedures than in thoracic or cervical surgeries. A large series of 2052 spinal surgeries reported only 17 cases (0.83%) of delayed CSF leakage. However, this incidence can reach up to 21% in revision surgeries.^[10]^

Commonly cited causes include severe spinal stenosis, dural adhesions to surrounding tissues, sharp bony fragments or spurs at decompression margins, and forceful events, such as violent coughing during emergence from anesthesia, straining during defecation, or urination.^[10]^ In this case, although no sharp bone spurs were noted at the decompression margin, the presence of a floating lamina was considered a key factor. It is hypothesized that, after ambulation, the mobile residual lamina shifts anteriorly, irritates repeatedly, and ultimately punctures the dura. This the novel reason of delay CSF differ from other reasons. Although an interspinous ligament tightening procedure was performed during the initial surgery to stabilize the lamina, this nonrigid fixation was insufficient to prevent dural penetration.

Clinically, vigilance is required for comorbidities in lumbar patients, which may influence the risk of DT. Hanna et al, reported on 439,220 lumbar fusion patients (11,636 with CSF leaks), identified through multivariate regression that advanced age (OR: 1.025, CI: 1.02–1.03), posterior approach surgery (OR: 1.71, CI: 1.59–1.78), chronic anemia (OR: 1.21, CI: 1.14–1.30), coagulopathy (OR: 1.40, CI: 1.24–1.58), obesity (OR: 1.22, CI: 1.15–1.29), cardiovascular disease (OR: 1.44, CI: 1.18–1.75), and electrolyte imbalance (OR: 1.53, CI: 1.44–1.63) were associated with a higher probability of CSF leak.^[14]^ Haft et al reported that variations in preoperative hemoglobin levels correlate with complications and surgical site infection rates after single-level lumbar fusion.^[15]^ This paper reports a special cause of DT, which is caused by the structure of the lumbar spine itself and iatrogenic operation, which is different from previous literatures.^[16,17]^

Current management strategies for postoperative DT and CSF leaks in lumbar surgery are categorized as either conservative or surgical. Regarding conservative management, Kalidindi et al reported successful treatment of 6 cases of late-presenting CSF leaks using bedside debridement, regular dressing changes, and antibiotics.^[18]^ Hanna et al reported that 79.9% of 11,636 patients with CSF leak underwent surgical management, including epidural patching, simple repair, and other surgical interventions.^[19]^ Surgical repair options include direct and indirect methods.^[20]^ Literature reports the use of muscle flap patches to cover dural defects when direct suturing is impossible.^[16,17,20]^ For instance, Lien et al employed a rotated sternocleidomastoid muscle flap to manage CSF leak after anterior cervical fusion.^[16]^ Policicchio et al described the use of pedicled multifidus muscle flaps to treat DT from penetrating spinal stab wounds in 2 cases.^[21]^ Wang et al reported a “sandwich” technique using flowable gelatin and gelatin sponge to repair DT.^[17]^ Additionally, indirect repair using polyethylene glycol (PEG) hydrogel sealants and epidural blood patching are viable options.^[22]^ Subarachnoid drainage and abdominal drainage are also effective approaches.^[23]^ Current evidence suggests no significant difference between direct and indirect repair with regard to postoperative bed rest duration or infection rates, making it unclear which method is superior.^[24]^ Compared to conservative management, surgical repair is associated with a shorter bed rest duration but shows no difference in hospital costs or mortality.^[19]^ The case was directly sutured by operation and achieved good clinical effect. Beyond specific repair techniques, strict bed rest is a crucial adjunct measure.^[9]^ Immobilization is vital for the management of CSF leaks. Currently, there is no definitive protocol for managing delayed CSF leaks, and treatment relies on the experience drawn from limited case reports.

The patient reported here, who presented with a floating lamina, highlights the need for preoperative vigilance regarding the risk of delayed CSF leak post-decompression. This case underscores the importance of meticulous preoperative imaging assessment and adoption of comprehensive and tailored surgical approaches. In some cases, upgrading the surgical plan beyond the conventional methods is necessary. Therefore, effective prevention is paramount.^[19]^ When symptoms of low CSF pressure arise postoperatively, prompt identification of the cause and suspicion of delayed CSF leak are critical. Managing a CSF leak requires utilizing the repair method with which the surgeon is most proficient, coupled with vigilant postoperative monitoring of drain output and patient symptoms, and readiness to implement further treatment strategies, if needed. This patient’s positive outcome was also facilitated by excellent compliance and cooperation, demonstrating the importance of a strong physician–patient relationship. For future similar cases, decompression should not retain mobile bony structures that could damage the dura and cause delayed leaks.

This study presents a single case report with limited follow-up. The described diagnostic and therapeutic approaches may have been influenced by the specific resources and expertise available at the treatment hospital and to the physicians involved. The report also reflects our lag and delay in dealing with some rare complications and special situations. There are potential limitations and challenges. This represents a limitation of this report, and the findings should be interpreted within this context. We believe the generalizability of the conclusions should be further emphasized to avoid over interpretation. More applicability of evidence will be reported like this.^[25,26]^ Moreover, lack of long-term imaging data is a pity.

## 4. Conclusion

Delayed CSF leaks after lumbar surgery are rare. This case resulted from incomplete preoperative assessment of the unstable lamina and inadequate intraoperative stabilization. It offers a valuable lesson for surgeons, emphasizing that preventing DT remains a critical skill in lumbar fusion.

## Acknowledgments

We are also grateful to all the researchers, including the physicians, nurses, and students, who participated in this study.

## Author contributions

**Conceptualization**: Liqiang Dong.

**Investigation**: Han Zhang, Bing Wu.

**Methodology**: Zhongcheng An.

**Software**: Junwei Feng.

**Visualization**: Binbin Tang.

**Writing** – **original draft**: Binbin Tang.

**Writing** – **review & editing**: Binbin Tang, Chen Chen, Zhongcheng An.
